# *In vivo* function of galectin-3 in motile cilia of airway epithelium

**DOI:** 10.1186/2046-2530-1-S1-P84

**Published:** 2012-11-16

**Authors:** D Delacour, T Piolot, E Pichard, V Contremoulins, T Dang, F Poirier

**Affiliations:** 1 Institut Jacques Monod CNRS Paris Diderot, France

## 

Galectins, a family of beta-galactoside binding lectins, participate in an exceptionally broad range of biological processes. It has been established that galectin-3 localizes at the primary cilium in epithelial cells, and that the absence of galectin-3 leads to major growth defects in primary cilia. We have now extended these original observations by studying the consequences of galectin-3 null mutation on the biogenesis and function of motile cilia *in vivo*. Using confocal and electron microscopy, we show that endogenous galectin-3 is also located in the motile cilia of adult mouse tracheas. Ultrastructural studies reveal that the absence of galectin-3 leads to a large panel of cilium abnormalities, including swollen cilia, compound cilia, and also abnormal axonemal organization (deviations from the expected 9+2 microtubular organization). We also monitored ciliary beat patterns by high speed videomicroscopy . These experiments revealed that, although beating frequency is unchanged, the amplitude of the movement was drastically reduced in *gal3-/-* tracheas. By following the dynamics of fluorescent beads applied to tracheal explants, we could demonstrate that the coordinated movement of the beads was severely affected in *gal3-/-* tracheas, in comparison to wt tracheas. Collectively, these data establish that galectin-3 is required for correct biogenesis and function of motile cilia in adult mice. Despite these defects, *gal3* mutant mice seem healthy in SPF animal house conditions. However, histological analyses and scanning electron microscopy revealed aberrant accumulation of mucus layers inside *gal3-/-* tracheas, suggesting the mice suffer from insufficient mucociliary clearance.

